# Pharmacology as a foreign language: A preliminary evaluation of podcasting as a supplementary learning tool for non-medical prescribing students

**DOI:** 10.1186/1472-6920-9-74

**Published:** 2009-12-18

**Authors:** Oonagh Meade, Dianne Bowskill, Joanne S Lymn

**Affiliations:** 1 School of Nursing, Midwifery & Physiotherapy, University of Nottingham, Queens Medical Centre, Nottingham, UK

## Abstract

**Background:**

Nurses and other health professionals in the U.K. can gain similar prescribing rights to doctors by undertaking a non-medical prescribing course. Non-medical prescribing students must have a thorough understanding of the pharmacology of prescribing to ensure safe practice. Pharmacology education at this level is complicated by the variation in students' prior subject knowledge of, and anxiety about, the subject. The recent advances in technology, particularly the potential for mobile learning, provide increased opportunities for students to familiarise themselves with lecture materials and hence promote understanding. The objective of this study was therefore to evaluate both the subjective (student perception) and objective (student use and exam results) usefulness of podcasts of pharmacology lectures which were provided as an extra learning tool to two cohorts (n = 69) of non-medical prescribing students.

**Methods:**

The podcasts were made available to students through the virtual learning environment WebCT. Use of podcasts by two successive cohorts of nurse prescribing students (n = 69) was tracked through WebCT. Survey data, which was collected from 44 of these students, investigated patterns of/reasons for podcast use and perceived usefulness of podcasts as a learning tool. Of these 69 students, 64 completed the pharmacology exam. In order to examine any impact of podcasts on student knowledge, their exam results were compared with those of two historical cohorts who did not have access to podcasts (n = 70).

**Results:**

WebCT tracking showed that 91% of students accessed at least one podcast. 93% of students used the podcasts to revisit a lecture, 85% used podcasts for revision, and 61% used the podcasts when they had a specific question. Only 22% used the podcasts because they had missed a pharmacology session. Most students (81%) generally listened to the entire podcast rather than specific sections and most (73%) used them while referring to their lecture handouts. The majority of students found the podcasts helpful as a learning tool, as a revision aid and in promoting their understanding of the subject. Evaluation of the range of marks obtained, mode mark and mean mark suggested improved knowledge in students with access to podcasts compared to historical cohorts of students who did not have access to pharmacology podcasts.

**Conclusions:**

The results of this study suggest that non-medical prescribing students utilised podcasts of pharmacology lectures, and have found the availability of these podcasts helpful for their learning. Exam results indicate that the availability of podcasts was also associated with improved exam performance.

## Background

In the U.K. there has been a gradual expansion of the incorporation of non-medical prescribing (NMP) within the health care system. The range of health care professionals to whom prescribing privileges have been extended include nurses, pharmacists, physiotherapists, radiographers and podiatrists [1]. While all of these groups of health professionals can train to become supplementary prescribers, prescribing in partnership with a doctor [2], nurses and pharmacists also have independent prescribing rights allowing them access to almost the same formulary of drugs as doctors [3].

In order to practice as non-medical prescribers, completion of an accredited NMP course at a Higher Education Institute is required. Pharmacology is one of the eight core components of the NMP course [4,5], and is recognised as being important in terms of patient safety by both educators and students alike [6-9]. Indeed, prescribing has been recognised by the medical profession as being an essential skill which is underpinned by a sound knowledge of the principles of pharmacology and therapeutics [10].

While pharmacists have a thorough knowledge of pharmacology gained from their pre-registration training they require education in relation to the clinical examination and consultation skills required for non-medical prescribing [11]. The same cannot be said of nurses, however, who are experienced in clinical examination and patient consultation but whose training has moved away from the biological to the social model of care [12]. Indeed, pharmacology has been specifically identified as an area of weakness in nursing education, both in the U.K. and elsewhere [7,8,13,14]. The implications of this for NMP education and ultimately patient safety are profound [15]. In terms of pharmacology education in NMP, our aim should be to encourage students not just to memorise enough pharmacology to pass the examination but to assimilate this knowledge and be able to integrate it into clinical practice.

Pharmacology education in NMP, however, can be complicated by a number of factors, including the range of academic levels and capabilities of students accessing such courses [9]. While pharmacists wishing to register on a NMP course must be educated to a degree level, there is no such prerequisite for nurses. According to a study performed by Tyler and Hicks [16], only 20% of nurses accessing prescribing courses would be accepted for training if these eligibility criteria were enforced. The situation is further complicated by the fact that approximately 50% of students accessing NMP courses have no more than a GCSE level qualification in a biological science [17]. Given the variation in student knowledge of biological and specifically pharmacological concepts at the beginning of the NMP course, and the limited number of teaching days on the course (n = 26), the communication of the necessary depth and breadth of pharmacology topics to this diverse group of students can be a significant challenge.

The availability of rapidly advancing technology has opened up a number of possibilities in relation to the teaching and learning environment available to students today. While reusable learning objects (RLOs) have been used to promote pharmacological understanding in nurses undertaking prescribing education, with some success [17], these tools are time-consuming and expensive to produce. This, coupled with many students' unfamiliarity with pharmacological terminology and concepts, suggests that podcasts may be a useful way of providing students with increased opportunities to familiarise themselves with lecture materials. As learning tools, podcasts usually constitute an audio recording of a lecture which can be listened to via a computer (at home or elsewhere) or downloaded to a portable media player such as an MP3 player or iPod [18-20]. Whilst some definitions of podcasts specify that media files are downloaded through web syndication (RSS fed) [21,22], a number of studies have used virtual learning environments to house podcasts [23-25]. Indeed, relating to the use of an RSS feed, Dale states that 'Alternatively, and within an educational context, the podcast could be uploaded onto a virtual learning environment (VLE) for students to listen to' [[22], pg 50] while Wolff argues that 'while the process hasn't been strictly that of a podcast, the end result is the same' [[23], pg 416].

The potential advantages of academic podcasts include: the proliferation of MP3 players or iPods among students, the ease of recording any lectures which have limited student involvement, the provision of distance learning opportunities and adding to existing course materials. Conversely, the potential disadvantages include: difficulties in searching through podcasts, recording multiple voices, concerns for classroom attendance, and the additional time requirements involved in recording, editing and producing the podcasts [26].

Reports of the use of podcasts in both undergraduate medical [27-29] and nurse education [30,31] in the U.K. and elsewhere are slowly beginning to emerge. Evaluative studies of the use of podcasts in medical education have indicated that podcasts helped students to learn their course materials and reduce their anxiety and stress relating to the subject [28] without impacting on classroom attendance [29]. Students found that podcasts positively impacted on their subject knowledge [29], and they considered podcasts to be useful supplementary learning tools [27]. Evaluation studies of the use of podcasts in other subject areas suggest that students rate podcasts as highly useful [24], find them helpful as review tools [32] and use podcasts to prepare for homework or exams [33]. Students also report using podcasts to enhance their understanding of complex parts of the lecture materials and to clarify the content of lectures [33]. While there is a growing literature on podcast use in a variety of academic settings, there is currently no data regarding the use of these learning tools for supporting pharmacology teaching, for use in NMP or for post-registration nurse education.

NMP students at the University of Nottingham are exposed to a degree-level pharmacology curriculum which cross-maps well with those of undergraduate medical curricula in pharmacology and therapeutics. Many of these students, however, arrive at University with limited biological science knowledge [17] and, perhaps unsurprisingly, struggle with this component of the course. Indeed, many students view pharmacology as a foreign language. Listening has been shown to be an effective educational tool, not least because the spoken word adds clarity and meaning, as well as communicating enthusiasm and thus stimulating motivation [34,35]. Indeed the cognitive theory of multimedia learning suggests that humans have distinct channels for processing both visual and auditory information and that combinations which include narration are effective in terms of knowledge transfer regardless of individual cognitive conditions [36]. In this current era of advanced technology, aural learning can even become mobile learning through the use of downloadable MP3 files. For all these reasons, this project aimed to utilise audio recordings of pharmacology lectures in an attempt to improve understanding of this subject in NMP students.

The purpose of this study was to provide an evaluation of the usefulness of podcasts of pharmacology lectures as a supplementary learning tool for NMP students. Specifically, the focus of this evaluation is on student use of the podcasts on offer, their perceptions of the usefulness of these podcasts and any potential impact on students' pharmacological knowledge.

## Methods

### Participants

All students attending the non-medical prescribing course at the University of Nottingham between September 2007 and September 2008 were part of this study.

The non-medical prescribing course is a six month course which has two intakes of students per year, September and January. Consequently this study involved students attending both the September 2007 course (n = 30) and the January 2008 course (n = 39). The concept of podcasts and information on where to find the podcasts within the virtual learning environment (WebCT) was explained to all students prior to the start of the course. This was achieved by an introductory session in a computer suite in which all students were logged on to the NMP course on WebCT and the available resources were demonstrated. Podcasts were explained to the students as being audio recordings of live lectures which could be accessed directly through the computer or downloaded to an MP3 player for mobile learning. This study had ethical approval from the University of Nottingham Medical School Ethics Committee.

### Production of podcasts

Seven key pharmacology lectures were recorded and made available as podcasts to two cohorts of non-medical prescribing students (Table 1). Lecture recordings were edited using the software programme 'Audacity' to improve the quality and flow of the full-lecture material. In order to make the lecture information more accessible to students, the audio recording was also divided up into bite-size chunks of lecture material, each containing one or two key concepts. Students were therefore able to listen to a portion of the recording which corresponded, for example, to slides 1-4 of the lecture handouts. Podcasts were uploaded onto the University streaming server and links were added into the appropriate section of the NMP course on WebCT. By clicking on a link on WebCT, students were able to stream podcasts directly or download them on to an MP3 player.

**Table 1:** Pharmacology lecture recordings made available to students

General principles of pharmacology
Pharmacokinetics 1 (absorption & distribution)

Pharmacokinetics 2 (metabolism & excretion)

Introduction to the autonomic nervous system

Analgesia

Anxiolytics & antidepressants

Contraception

The preparation of the podcasts initially required IT assistance from a member of the School of Nursing Educational Technology group and it took approximately four hours to produce one complete podcast and its associated subsections in MP3 format. As experience with the technology increased however, assistance was no longer required and a podcast could be produced in under two hours.

### Student use of pharmacology podcasts

Podcasts were made available to two consecutive cohorts of NMP students (n = 69). The podcasts accessed by students, the frequency of that access, and the average time spent accessing each podcast was measured using the tracking facility in WebCT.

### Student perceptions of podcast value

The assessment of student perceptions of podcast value involved the collection of questionnaire data from two successive cohorts of NMP students (n = 69) who had access to podcasts of seven key pharmacology lectures. This questionnaire consisted of 21 items, contained a mixture of both fixed and open-response questions and was divided into two sections.

Section one collected demographic details on age, gender and job title. Participants were also asked in this section about their comfort level with internet-based technology, whether they had home access to a computer with internet resources and whether they owned or had access to an MP3 player. Section two investigated both students' patterns of podcast use and their perceptions of the usefulness of the podcasts. Students were asked to document how many times they accessed each of the individual podcasts on a five point scale (options included; Never, 1-3 times, 4-6 times, 7-9 times and 10 times or more). Respondents were also asked to document how they accessed the podcasts (i.e. as an MP3 file for download to an MP3 player, or directly streamed via WebCT on the computer) and whether they generally used the podcasts while referring to the lecture handouts (slides) or without these. They were also asked whether they generally listened to the whole podcasts or listened to specific parts. In order to look at the usability of the podcasts, the survey asked students if they found the podcasts to be accessible, and easy to navigate through.

To gain feedback on students' reasons for using the podcasts, participants were asked to rate, on a scale of 1-5, their agreement with the following statements: "I used podcasts when I missed a session"; "I used podcasts when I had a specific question"; "I used podcasts when I wanted to revisit the lecture generally"; "I used podcasts when I needed to revise" and "I used podcasts for other reasons".

Students were also asked to rate their perception of the usefulness of the pharmacology podcasts as a learning tool, as a revision aid, and in terms of promoting understanding of course materials on a scale of 1-5 ranging from 'very useful' to 'very unhelpful'. Finally, participants were given the opportunity to provide further feedback on their experience of podcast use in response to the question "Do you have any further feedback about the use of podcasts which has not been explored in this questionnaire?"

The questionnaire was designed following discussion between the authors and was piloted with two students. No issues with regard to either content or face validity arose. Students were sent an invitation letter and questionnaire by post. The invitation letter explained to students that their participation was entirely voluntary, anonymous and confidential. Details were also given to students about how to contact the research team should they wish to remove their data from the study. The questionnaire package contained a pre-paid envelope for the return of completed surveys. After three weeks, reminder letters, containing a further copy of the questionnaire, were posted out to students.

Questionnaire data was entered into SPSS (Version 14.0) which was then used to generate frequencies and percentages of responses to each question.

### Exam Performance

In order to determine whether podcast provision had a measurable impact on student knowledge and understanding, exam results were compared between two cohorts of students who had access to podcasts (n = 64) and two previous cohorts who did not have access to podcasts in their course materials (n = 70). The number of students undertaking the exams (n = 64) was lower than the number of students who attended the course and had access to podcasts (n = 69) as a result of five students suspending their studies prior to the examination. In an attempt to control for potential confounding variables, only topics which were taught by the same lecturer and had the same learning outcomes were compared. The results of the two cohorts who had podcasts and the two cohorts who did not were compared on three topics: pharmacokinetics, autonomic nervous system and pharmacology of pain. Comparisons were made in relation to the range of scores achieved by students, the mode and the mean score achieved. Unpaired t-tests were used to detect differences in the mean scores of those who had access to the podcasts compared to those who did not.

## Results

### Tracking student podcast use

Of the 69 students who had access to the pharmacology podcasts, 63 had accessed at least one podcast. Thus, 91% of students overall accessed the podcasts. Students clicked on the links to the podcasts between 0 and 48 times. The percentage of students who accessed the podcasts was similar for cohort one (90%) and cohort two (92%). Tracking data indicated that the most accessed podcast was 'general principles' which was accessed by 54 students while 'contraception' was accessed the least, being used by 22 students. The average length of time students spent accessing the podcasts ranged from 25.06 to 61.9 minutes (Table 2), Student registers for the two cohorts of students demonstrated that a total of 10 students had missed a pharmacology session and WebCT tracking data indicated that 8 of these 10 students accessed the pharmacology podcast associated with the missed session(s).

**Table 2:** Frequency of individual podcast use.

Podcast	No of students accessing podcast (out of 69)	No of times accessed by student mean ± SEM	Time accessed by student (mins) mean ± SEM
General principles of pharmacology	54	4.2 ± 0.6	61.92 ± 10.93

Pharmacokinetics 1 (absorption & distribution)	44	3.3 ± 0.4	53.20 ± 8.10

Pharmacokinetics 2 (metabolism & excretion)	42	2.6 ± 0.3	48.69 ± 9.14

Introduction to the autonomic nervous system	47	5.5 ± 0.7	61.70 ± 12.02

Analgesia	27	2.0 ± 0.3	31.82 ± 7.97

Anxiolytics & antidepressants	26	2.4 ± 0.4	33.98 ± 9.35

Contraception	22	2.0 ± 0.2	25.06 ± 7.25

WebCT tracking data indicating the number of students who accessed each podcast, the average number of times each podcast was accessed by an individual student and the average length of time each podcast was accessed for.

### Survey of student use and perceptions of the podcasts

The total response rate for the questionnaire was 64% (44/69) and was not dissimilar for both of the cohorts (18/30 (60%) cohort one; 26/39 (67%) cohort two).

#### • Demographic characteristics

**Figure 1:**
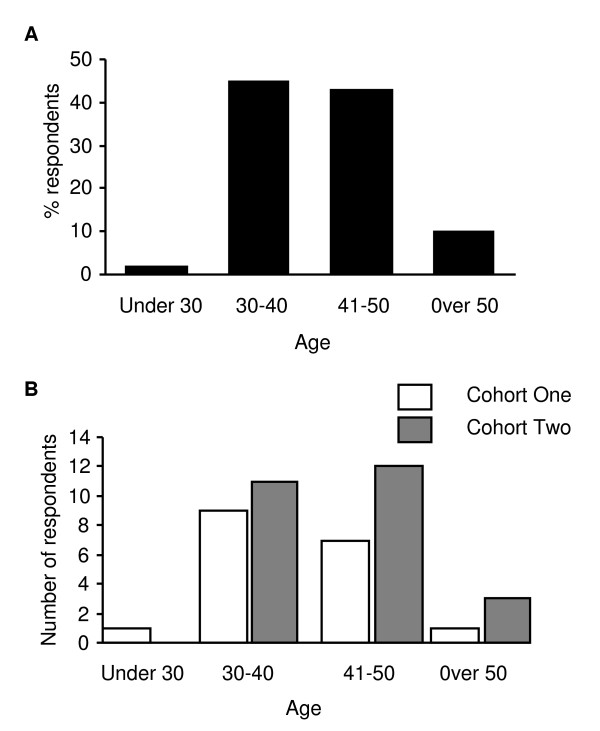
**Participant age demographics**. **A **- This graph demonstrates the age profile of students accessing the non-medical prescribing course who returned the postal questionnaire - combined data from two cohorts of students (n = 44). **B **- This graph demonstrates the age profile of students from two separate cohorts who returned the postal questionnaire. Cohort one n = 18. Cohort two n = 26.

**Figure 2:**
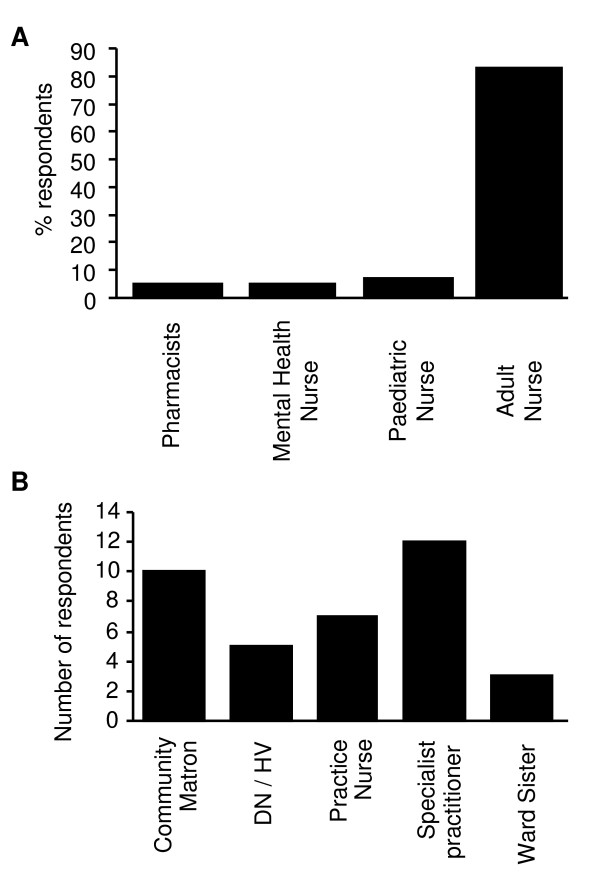
**Breakdown of non-medical prescribing students by profession**. **A **- This graph shows the breakdown of those students attending the non-medical prescribing course, who returned the postal questionnaire, by professional qualification - combined data from two cohorts of students (n = 44). **B **- This graph demonstrates the breakdown of registered adult branch nurses by role - combined data from two cohorts of students (n = 37).

Most respondents were in the 30-40 (n = 20) or the 41-50 (n = 19) age groups. One participant was under thirty years of age and four participants were aged over fifty years. The profile of the individual cohorts was similar (Figure 1). Of the total respondents, 84% (37/44) were female while 16% (7/44) were male. The majority of respondents were adult nurses (37/44) while a small number were mental health nurses (3/44), paediatric nurses (2/44) and pharmacists (2/44). The adult nurses could be grouped into community matrons, practice nurses, district nurses/health visitors, specialist nurses (including pain, diabetes, cardiac and intensive care) and ward sisters (Figure 2).

#### • Comfort with internet technology

All respondents had access to a home computer with internet access and most either owned or had access to an MP3 player/iPod (63%). In contrast to these data, only 42% rated their comfort levels with internet technology as being high or very high.

#### • Method of podcast access

Despite the fact that 63% of respondents had access to an MP3 player/iPod, most respondents indicated that they listened to the podcasts directly through their computer (81%). Only 7% downloaded the podcasts and listened to them exclusively on an MP3 player or iPod while a further 12% used a combination of both methods. The majority of students (78%) generally listened to the entire lecture rather than accessing specific sections. Similarly most students listened to podcasts whilst referring to their lecture slides (73%) rather than using them as a stand-alone learning tool. In relation to the accessibility of podcasts, 95% of respondents found the podcasts both accessible and easy to navigate through and 93% of respondents were able to find the answer to any specific questions they had. However, student feedback from open text boxes suggested that some students had difficulty downloading the podcast to their MP3 players:

"I found it difficult to download podcasts on to MP3 player, easy to access straight from WebCT."

#### • Reasons for podcast use

In terms of reasons for using the podcasts, the majority of respondents agreed or strongly agreed that they used podcasts when they wanted to generally revisit the lecture, specifically for revision purposes, and also when they had a specific question. A minority of respondents agreed or strongly agreed that they used podcasts when they missed a lecture session (Table 3). A third of the respondents had used the podcasts for reasons other than those outlined on the questionnaire and when asked in an open text box to clarify their reason for accessing the podcasts, one student replied:

**Table 3:** Reasons for student use of pharmacology podcasts.

Reason for use	% respondents agreed or strongly agreed
To revisit the lecture generally	93

Specifically for revision purposes	85

To answer a specific question	61

Missed session	22

Other	33

Self-reported reasons for use of pharmacology podcasts given by two cohorts of NMP students (n = 44)

"To confirm my understanding: I used it as a repetitive tool to familiarise myself with terminology I hadn't heard before - to promote my comfort - to then go on and digest the subject."

#### • Perceptions of podcasts

**Figure 3:**
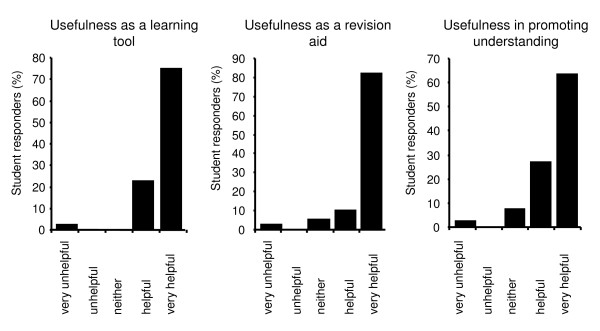
**Students perceptions of the usefulness of podcasts**. This graph represents student's perceptions (n = 44) of the usefulness of pharmacology podcasts as learning tools, revision aids and in promoting understanding. Students rated podcast usefulness on a 5-point likert scale with 1 being very helpful and 5 being very unhelpful.

In terms of students' perceptions of the usefulness of the pharmacology podcasts, the vast majority of respondents rated the podcasts as very helpful or helpful as a revision tool, as a learning tool, and as a tool for promoting their understanding of pharmacology (Figure 3). Students rated the helpfulness of the podcasts on a scale of 1-5, with very unhelpful representing a score of 1 through to very helpful which represented a score of 5. The mean score achieved for each of the three characteristics was 4.7 for both 'learning tool' and 'revision aid' and 4.5 for 'promoting understanding'.

Most respondents who provided open text comments regarding podcast use provided positive evaluations of their experiences of podcasts (93%):

"Absolutely brilliant as an assistant to the course content"

"An excellent learning tool to reinforce my understanding of the basics of dynamics, kinetics and the ANS. I would not have understood these subjects without revisiting the lectures time and again"

"I found they were extremely helpful in consolidating each subject following lectures. They helped info sink in!"

"I think they are a fantastic, useful tool"

"In general it was an excellent resource that really aided me in developing my knowledge"

"I found the podcasts to be a great benefit to accompany the course."

Similarly, 84% of respondents agreed that podcasts would be useful for other areas of the NMP course. When asked to document any further thoughts they had on the podcasts, some students expressed an interest in the incorporation of more podcasts into the pharmacology course or indeed in other subjects on the NMP course:

"I feel it would be good to have more of the pharmacology lectures put onto podcast e.g. cardiovascular, antimicrobials etc."

"Just wanted to say that all lectures should be made available in this format. It really was invaluable as a learning tool for busy people."

"Would have been useful for most of the lectures."

#### • Use of individual podcasts

**Figure 4:**
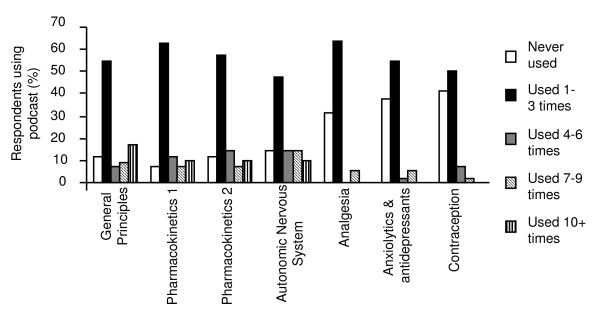
**Student access of individual pharmacology podcasts**. This graph represents the self-reported access profiles of specific pharmacology podcasts by two combined cohorts of non-medical prescribing students (n = 44).

All seven pharmacology podcasts were accessed by students, although the podcasts related to the basic pharmacological concepts (general principles, pharmacokinetics, autonomic nervous system) were accessed more frequently than those related to specific therapeutic areas (Figure 4).

#### • Barriers to podcast use

Some useful comments were also provided regarding the barriers to podcast use which students experienced. One participant described how "using a computer is not my preferred way of learning". Background noise also seemed to hinder the perceived quality of some podcasts: *"Some podcasts were quite noisy i.e. papers shuffling etc. which made it hard to concentrate"*. For one student who had a hearing impairment this background noise in the recordings took away from the potential usefulness of the podcasts:

"My negative feedback is simply because I was unable to hear the podcast as there was a lot of echoing - I'm sure if I didn't have hearing problems it would have been a very useful tool."

One further comment suggested that one student found that differences in lecturing style impacted on her ability to concentrate on particular podcasts.

" [lecturer A's]*were excellent*, [lecturer B's]*really good but *[lecturer C's]*were more difficult to concentrate on."*

### Exam Performance

There were two pharmacists in the group with access to podcasts (3%) and three in the group without access to podcasts (4%) thus the two groups were from comparable professional backgrounds.

In relation to markers of student knowledge the range of marks achieved for questions related to all of the three areas analysed (pharmacokinetics, autonomic nervous system, analgesia) were improved in the cohorts of students who had access to podcasts. Similarly, the mode mark achieved improved in relation to analgesia and the autonomic nervous system and was unchanged for pharmacokinetics.

**Figure 5:**
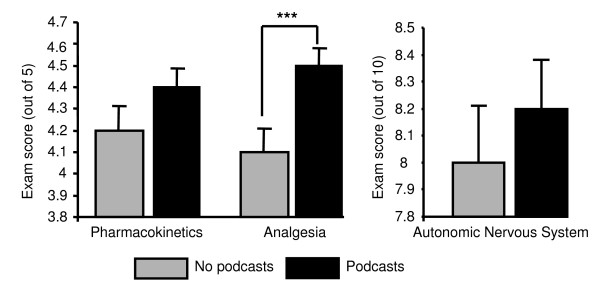
**Exam score comparisons between students who had access to podcasts and those who did not**. This graph represents student knowledge as measured by exam score from two historical cohorts of students who did not have access to podcasts (n = 70) compared to two cohorts who did have access to podcasts (n = 64). Exam questions took the form of stem & branch true/false and possible scores obtained ranged from 0-5 in whole numbers for pharmacokinetics and analgesia and 0-10 in whole numbers for the autonomic nervous system. Statistical comparison of exam scores from the two groups was performed using an unpaired t-test (*** = p < 0.001).

Students from the two cohorts who had access to the pharmacology podcasts also exhibited higher mean exam scores in relation to all three topics with the improvement in the mean score for analgesia being statistically significant (Table 4, Figure 5).

**Table 4:** Comparison of exam performance of cohorts with and without access to podcasts.

		Range of Marks	Mode	Mean (± SEM)	P value
**Pharmacokinetics**	No Podcasts	1-5	5	4.2 ± 0.11	0.059
(marked out of 5)	Podcast	2-5	5	4.4 ± 0.09	
**Autonomic**	No Podcasts	2-10	8	8.0 ± 0.21	0.173
**Nervous System** (marked out of 10)	Podcast	3-10	9	8.2 ± 0.18	
**Analgesia**	No Podcasts	1-5	4	4.1 ± 0.11	0.001
(marked out of 5)	Podcast	2-5	5	4.5 ± 0.08	

Comparison of the range of exam scores, mode exam score and mean exam score obtained by two cohorts of NMP students who had access to pharmacology podcasts (n = 64) and the two previous cohorts of students who did not have access to pharmacology podcasts (n = 70). Statistical comparison of mean exam scores obtained between the two groups of students was performed by t-test.

## Discussion

The purpose of this study was to evaluate the usefulness of podcasts as a supplementary learning tool for NMP students. As the production of podcasts is initially a time consuming process, an important aspect of the current evaluation was to determine actual levels of use of the available podcasts. Tracking data from this study demonstrated that 91% of students accessed these audio recordings. This rate of use is high compared to rates reported for marine science and medical students in both the U.K. and Germany respectively [24,27,29]. This increased uptake of podcasts compared to other studies may be a result of the differing nature of the student population. Previous studies have involved undergraduate students, whereas this study involved post-registration students who are studying part-time alongside working in senior positions and are of the age group that may have dependent relatives. Moreover some of these students are employed in positions which require them to obtain a NMP qualification as part of their job description (e.g. Community Matrons) and risk losing their job if they fail to obtain this qualification. Taking these factors into consideration, as well as with the lack of biological science background knowledge, it is possible that this population of students may be more inclined to take their studies very seriously and explore all available learning tools in order to reach a successful outcome. Additionally the increased uptake may be the result of the initial orientation session run for the students detailing how to access the podcasts. The importance of orientation/explanation for improving scientific understanding from multimedia learning has previously been demonstrated [37,38].

Despite the availability of downloadable files, the majority of students accessed the podcasts directly through the computer. While this may be related to the nature of the student population and their self-confessed lack of familiarity with internet technology, these data are not dissimilar to results from other studies [24,25] which have used much younger groups of students, suggesting that the underlying reasons for this may be more complex than initially thought. It was clear, however, that housing the links to the downloadable MP3 files in WebCT did present some technical problems for students and this was addressed in the form of a question and answer session with an information technology technician over the lunch period on one occasion.

In terms of the reasons for podcast use, and student perceptions of their usefulness, the data shown here are consistent with the published data from other student groups in terms of their usefulness as both a learning tool [24], and for revision [25,32]. The results of this study are also consistent with existing research on the use of podcasts with medical students, which also found that podcasts were useful supplementary learning tools [27] that had a beneficial effect on student knowledge [29].

In relation to the present study, it should be highlighted that, of the three students who rated the podcasts as 'neither helpful nor unhelpful' as a revision aid and at promoting understanding, two were pharmacists while the other was a student who had a PhD. It is likely then, that none of these students required additional support in relation to their pharmacology knowledge. One student who had a hearing impairment also rated the podcasts as unhelpful (see section on "Barriers to podcast use"). This does raise the important issue of using live lecture recordings versus studio recordings. While it has been suggested that podcasts may be useful tools for students with learning difficulties and those whose first language is not English [22,39], it may be that studio recordings of lecture material is more appropriate. On the other hand, if the aim is simply to provide a supplementary learning tool for all students, the live lecture recordings should be sufficient and involve considerably less time commitment.

The potential impact of podcast availability on student attendance at lectures has been raised in previous research [28,29,32,39,40]. Attendance was not an issue in this study as registers are taken twice a day and students are required to attend a minimum of 80% of sessions. Failure to attend 80% of the taught sessions results in students being back-grouped. While these criteria may account for the low levels of students reporting that they used podcasts when they missed a pharmacology session, there is also evidence that the availability of podcasts in medical, and other undergraduate, education may not significantly impact on attendance [26,29,39]. There are, however, instances where students cannot attend sessions and previously they would have had to work solely from textbooks to access the required knowledge. Indeed, WebCT tracking data from this study revealed that 80% of the students who missed a session accessed the associated lecture recording. Arguably, the introduction of podcasts allows students easier access to the requisite information and may relieve anxiety [28].

The WebCT tracking data correlated well with the questionnaire data regarding student use of podcasts with both sources indicating that all the pharmacology podcasts were accessed by the students, although the profile of access differed. The podcasts concerned with basic concepts were more heavily used (accessed by more students, more frequently and for a longer average period of time) than those related to specific therapeutic areas. This may be the result of students feeling more comfortable with the therapeutics, as a result of their clinical experience, than with the basic concepts. Alternatively, it should be considered that the basic concept lectures were all taught by a single lecturer, while each therapeutic session was taught by a different lecturer, therefore raising the issue of lecturing style in relation to successful podcasts. As is evidenced in the 'Barriers to Podcast Use' section above, one student found it more difficult to concentrate on some lecturer's podcasts compared to others. A clear understanding of what makes a successful podcast may or may not include the same criteria as a successful lecture and this is an area in which research input would be invaluable.

The high percentage of students who would like to see the use of podcasts expanded is again consistent with literature from other areas [24]. Indeed, the pharmacists in this study were positive about the use of these tools to support other areas of the course: *"For pharmacists, pharmacology podcasts less helpful than for nurses. Some stuff on ethics and examinations would be helpful*."

While evaluation of student perceptions of podcasts makes up the majority of the available literature [24,25,27,28,32], there is little available data in terms of a measurable impact of podcast use on student knowledge levels. In this study, we found a consistent improvement in the mean exam score in cohorts of students who had access to podcasts compared to those who did not. While this was only statistically significant for analgesia, the remaining areas (pharmacokinetics, autonomic nervous system) did show different ranges of scores achieved by students and the mode score was increased for both analgesia and the autonomic nervous system. The way these areas are examined is by means of stem and branch true-false questions (concerned with knowledge application rather than recall of exact phrases or definitions) and as such students can only achieve whole marks. This, coupled with the numbers of students involved, might make it difficult to clearly detect modest effects on student learning statistically, but these data are suggestive of such an improvement. The similarity in professional make-up of the comparative groups makes it unlikely that the improvement in exam responses was the result of an imbalance in the number of pharmacists, and thus background pharmacological understanding, among the two groups. This suggests that the improved knowledge may indeed be a result of the availability of pharmacology podcasts improving nurses' confidence with basic terminology and concepts.

The limitations of this study are essentially based around the small number of students involved and the fact that this study was conducted with a very specific group of students and may not therefore be applicable to other student groups. This study was also concerned with only pharmacology podcasts and there is no guarantee that similar results would be achieved in other subjects even within the same student group. The questionnaire format of this study also limits the nature of the data obtained and a more detailed study of barriers and facilitators to podcast use in this student group would provide important information which may impact on the use of these tools to stimulate learning.

## Conclusions

The aim of this study was to evaluate the usefulness of pharmacology podcasts as a supplementary learning tool for NMP students. Tracking data indicated that the pharmacology podcasts were heavily accessed by non-medical prescribing students. Questionnaire data indicated that students rated the podcasts as being useful or very useful in terms of a learning tool, revision aid and in promoting understanding. A comparison of exam results between students who had access to podcasts and historical cohorts of students who did not, supported students' positive perceptions of the usefulness of podcasts in promoting pharmacology understanding. Whilst lecture recordings were available as downloadable MP3 files, most students chose to access these tools directly through their PC and hence the opportunity for mobile learning was not taken. Overall the results of this pilot study were extremely positive and support the further provision of pharmacology podcasts in future.

## Competing interests

The authors declare that they have no competing interests.

## Authors' contributions

JSL and DB conceived of, designed the study and obtained the funding. JSL and OM acquired the questionnaire data. OM analysed the data. JSL performed statistical analysis of the data. OM drafted the manuscript. All authors have read and approved the final manuscript.

## Pre-publication history

The pre-publication history for this paper can be accessed here:

http://www.biomedcentral.com/1472-6920/9/74/prepub
